# The Thermodynamics of Medial Vascular Calcification

**DOI:** 10.3389/fcell.2021.633465

**Published:** 2021-04-14

**Authors:** Ángel Millán, Peter Lanzer, Víctor Sorribas

**Affiliations:** ^1^Instituto de Nanociencia y Materiales de Aragón (INMA), CSIC-Universidad de Zaragoza, Zaragoza, Spain; ^2^Division of Cardiovascular Disease, Department of Internal Medicine, Health Care Center Bitterfeld, Bitterfeld-Wolfen gGmbH, Bitterfeld-Wolfen, Germany; ^3^Molecular Toxicology Group, Department of Biochemistry and Molecular and Cell Biology, University of Zaragoza, Zaragoza, Spain

**Keywords:** medial vascular calcifications, thermodynamics, calcium and phosphate homeostasis, ectopic calcification, mineralization

## Abstract

Medial vascular calcification (MVC) is a degenerative process that involves the deposition of calcium in the arteries, with a high prevalence in chronic kidney disease (CKD), diabetes, and aging. Calcification is the process of precipitation largely of calcium phosphate, governed by the laws of thermodynamics that should be acknowledged in studies of this disease. Amorphous calcium phosphate (ACP) is the key constituent of early calcifications, mainly composed of Ca^2+^ and PO_4_^3–^ ions, which over time transform into hydroxyapatite (HAP) crystals. The supersaturation of ACP related to Ca^2+^ and PO_4_^3–^ activities establishes the risk of MVC, which can be modulated by the presence of promoter and inhibitor biomolecules. According to the thermodynamic parameters, the process of MVC implies: (i) an increase in Ca^2+^ and PO_4_^3–^ activities (rather than concentrations) exceeding the solubility product at the precipitating sites in the media; (ii) focally impaired equilibrium between promoter and inhibitor biomolecules; and (iii) the progression of HAP crystallization associated with nominal irreversibility of the process, even when the levels of Ca^2+^ and PO_4_^3–^ ions return to normal. Thus, physical-chemical processes in the media are fundamental to understanding MVC and represent the most critical factor for treatments’ considerations. Any pathogenetical proposal must therefore comply with the laws of thermodynamics and their expression within the medial layer.

## Introduction

Medial vascular calcification (MVC) represents a chronic, progressive disorder of calcium metabolism, eventually resulting in the formation of calcium deposits, already described by Virchow (1858). MVC is an important predictor of cardiovascular morbidity and mortality in patients with chronic kidney disease (CKD) or diabetes mellitus (DM) (Niskanen et al., 1994; Lehto et al., 1996; London et al., 2003; Aitken et al., 2012; Vlachopoulos et al., 2015). This is partially due to the persistent effects of MVC, such as increased arterial wall rigidity, systolic afterload of the left ventricle, and pulsatility (Vlachopoulos et al., 2010; Chirinos et al., 2019), as well as interference with arterial remodeling (Fok and Lanzer, 2018). MVC is also present in aging (Pescatore et al., 2019) and in a number of less common diseases, including pseudoxanthoma elasticum (Lefthériotis et al., 2013; Devriese et al., 2019); rheumatoid arthritis (Paccou et al., 2012); β-thalassemia (Aessopos et al., 1998); calciphylaxis (Seethapathy and Noureddine, 2019); Kawasaki disease (McCrindle et al., 2017); Singleton–Merten syndrome (Feigenbaum et al., 2013); secondary hyperparathyroidism (Terai et al., 2009); vitamin K deficiency (Shea and Booth, 2019), including warfarin administration (Poterucha and Goldhaber, 2016) and vitamin D metabolic disorders (Wang et al., 2018); Generalized Arterial Calcification of Infancy (GACI) (Rutsch et al., 2003); Arterial Calcification due to CD73 Deficiency (ACDC) (St Hilaire et al., 2011); and Idiopathic Basal Ganglia Calcification (IBGC) (Wang et al., 2012).

Despite extensive research on the mechanisms of MVC over the past 20 years, our understanding of vascular calcifications (VC) pathogenesis remains incomplete (Johnson et al., 2006; Stabley and Towler, 2017; Disthabanchong and Srisuwarn, 2019), and no therapy based on the etiology of MVC is available (Ruderman et al., 2018; Schantl et al., 2019), with the exception of a few experimental/preliminary data on restraining calcifications in CKD (Mendoza et al., 2008; Finch et al., 2013; Finer et al., 2014; Leibrock et al., 2016; Díaz-Tocados et al., 2017; Lang et al., 2018; Perelló et al., 2018). To date, a number of pathogenetic mechanisms of VC have been proposed and are reviewed in the literature (Sage et al., 2010; Durham et al., 2018; Rogers and Aikawa, 2019). In the aging, for example, both the accumulation of the nuclear protein, progerin (pre-Lamin A), and the disruption of nuclear lamina are correlated with MVC (Liu et al., 2013). In diabetes, the correlation occurs with the accumulation and binding of advanced glycation end-products (AGEs) to the lysine residues in elastin and collagen (Bruel and Oxlund, 1996; Conway et al., 2010). Under uremic conditions, the kidney releases injury factors into the blood flow, such as WNT inhibitors (Dkk1 or sclerostin) or activin A, which precedes the increase of alkaline phosphatase expression (Fang et al., 2014; Hortells et al., 2017). Heart activity and hemodynamics cause mechanical stress, the expression of Bmp2, and oxidative stress in vascular smooth muscle cells (VSMC) (Rutkovskiy et al., 2019). Reactive oxygen species induce the expression of Runx2 (Chang H. B. et al., 2008), and apoptotic bodies also contribute to MVC (Proudfoot et al., 2000). Damaged cells can also release elastin fragments, which increase the number of nucleation sites (Wanga et al., 2017) upon binding to the extracellular matrix (ECM). Membrane-bound matrix vesicles called exosomes behave like apoptotic bodies and seem to be released from VSMC (Kapustin et al., 2015). The degradation of elastin by matrix metalloproteinases increases the affinity for calcium ions and activates the transforming growth factor-ß signaling pathway (Pai et al., 2011).

From the aforementioned examples of conditions of unrelated diseases, in which MVC is only a part of the phenotype, it appears likely that different pathogenetical mechanisms will eventually result in ectopic VC. However, despite of these pathogenetical differences all of these diseases will have in common the processes of nucleation, precipitation, and crystallization of calcium phosphate deposits that follow the same physical processes governed by the laws of thermodynamics. Therefore, due to the universal validity and fundamental importance of these laws, any scientific theory of MVC pathogenesis, experimental model and even treatment’s proposal must ultimately comply with them. Thus, the compliance of experimental data with the laws of thermodynamics should be considered the litmus test of the data’s scientific validity. For example, in the case of CKD, hyperphosphatemia has been considered to play a predominant role as a cause of calcium precipitation and MVC, but thermodynamics reveals (see below) that the plasma phosphate concentrations observed in CKD do not suffice to cause calcium precipitation under these conditions, and even less in healthy human beings.

Here, we shall review the principles of biophysics of calcium deposition and the laws governing bio-mineralization, including the roles of promoters and inhibitors in the initiation and growth of calcification deposits. While this review may not provide definitive answers regarding the pathogenesis of MVC, it aims to establish the ground rules for studies on MVC pathogenesis.

## The Fundamental Thermodynamics of Calcium-Phosphate Precipitation

### Key Concepts

There are key facts in calcification that derive straightforward from the laws of thermodynamics, and therefore must always be obeyed. The most basic concept is the second law of thermodynamics, stating that the entropy of an isolated system can never decrease (see Box 1). This law sets forth the condition for spontaneous chemical processes governed by the Gibbs expression (Gibbs, 1874–1878): D*G* < 0, where D*G* is the variation of the Gibbs free energy, which it is related to the entropy change. In this review we use often the term *precipitation* concerning the process of calcium phosphate solid formation from its free ions in a solution state. With this term we refer to its classic definition: “a relatively rapid formation of a sparingly soluble crystalline—or sometimes amorphous—solid phase from a liquid solution phase” (Karpiński and Jerzy Bałdyga, 2019). Thus, translating the Gibbs condition to the precipitation of ions from solutions or biological matrices, it turns out that the activity product of free ions in solution must by higher than the corresponding activity in the solid. This relationship, expressed in terms of supersaturation (S), characterizes the thermodynamic conditions for all spontaneous calcifications as *S* > 1 (Myerson et al., 2019), with no exceptions. Thus, this expression means that the precipitate will be formed only if the product of the activities of free ions in the fluid state is higher than the product of the activities of ions in the solid state given by **the solubility product, K_*sp*_**; when *S* < 1, the solid will dissolve. Thus, the key point for determining whether MVC is likely to occur is to determine the activity of the free ions in the tissues that compose the medial layer. However, this is not a trivial task, given that there are many factors that affect the activities of free ions in a fluid, such as ionic strength, phosphate proton dissociation equilibria, the sequestration of free ions by ligands, the precipitation of different calcium phosphate solids, and the viscosity of the medium. Due to such high level of biological complexity, the available values of K_*sp*_ and other related thermodynamic constants needed for these calculations that have been derived from in-vitro conditions may be only approximates. In addition, it is important to realize that the thermodynamic condition for solid growth is related to ionic activities and not the ionic concentration. While the latter can be determined by chemical analysis, the former are far more difficult to determine due to the biological and the structural complexity of the medial layer. Concentrations and activities are related by the activity coefficient, γ(*a* = γ⋅ *conc*), which, in turn, is related to the **ionic strength, *I*** (see Box 2 and Table 1).

**BOX 1. The driving force of precipitation.**

Similar to all chemical processes, the precipitation of calcium phosphates is governed by the Gibbs free energy of the component ions in the solid and the solution.^1^ A chemical system is at equilibrium when the Gibbs free energy is zero.

(1) △⁢G=0

Δ*G* is given by:

(2) $$△\,G=V\,△\,P-S\,△\,T+\sum \mu_{i}\,△\,n_{i}$$

which at constant pressure and temperature is reduced to:

(3) $$△\,G=\sum \mu_{i}\,△\,n_{i}$$

where μ_*i*_ is the chemical potential of component *i*, and *n*_*i*_ is its number of moles. The chemical potential is defined as:

(4) $$\mu_{i}=\mu_{i}^{0}+R\,T\,l\,n\,a_{i}$$

where *a*_*i*_ is the activity of component *i*.

For a binary solid in equilibrium with its component ions in aqueous solution.

(5) A⁢(a⁢q)+B⁢(a⁢q)⇔A⁢B⁢(s⁢o⁢l⁢i⁢d)

the equilibrium condition would be:

(6) $$△\,G=△\,G_{0}+R\,T\,l\,n\,a_{A\,B}\,\left(s\,o\,l\,i\,d\right)-R\,T\,l\,n\,a_{A_{i}}\,\left(a\,q\right)+R\,T\,l\,n\,a_{B_{i}}\,\left(a\,q\right)=0$$

considering that the activity of a solid is zero, the equation is reduced to:

(7) $$△\,G=△\,G_{0}-R\,T\,l\,n\,\left[a_{A_{e}}\,\left(a\,q\right)\cdot a_{B_{e}}\,\left(a\,q\right)\right]=0$$

and

(8) $$△\,G_{0}=R\,T\,l\,n\,\left[a_{A_{e}}\,\left(a\,q\right)\cdot a_{B_{e}}\,\left(a\,q\right)\right]$$

From that expression, the solubility product is defined as

(9) $$K_{s\,p}=a_{A_{e}}\,\left(a\,q\right)\cdot a_{B_{e}}\,\left(a\,q\right)$$

*K*_*sp*_ is a constant that varies with the temperature as:

(10) $$K_{s\,p}=e^{\frac{-△\,G_{0}}{R\,T}}$$

For a crystal to grow from a solution:

(11) $$△\,G=△\,G_{0}-R\,T\,l\,n\,\left[a_{A_{i}}\,\left(a\,q\right)\cdot a_{B_{i}}\,\left(a\,q\right)\right]<0$$

(12) $$△\,G=R\,T\,l\,n\,\left(K_{s\,p}\right)-R\,T\,l\,n\,\left[a_{A_{i}}\,\left(a\,q\right)\cdot a_{B_{i}}\,\left(a\,q\right)\right]<0$$

and

(13) $$R\,T\,l\,n\,\left[\frac{a_{A_{i}}\,\left(a\,q\right)\cdot a_{B_{i}}\,\left(a\,q\right)}{K_{s\,p}}\right]>0$$

From that expression, the ionic activity product is defined as

(14) $$I\,A\,P=a_{A_{i}}\,\left(a\,q\right)\cdot a_{B_{i}}\,\left(a\,q\right)$$

and supersaturation as

(15) $$S=\left(\frac{I\,A\,P}{K_{s\,p}}\right)$$

Thus, a solution would be supersaturated with respect to a given solid when *S* > 1.

*S* is the driving force for crystallization, and it is the key factor in precipitation processes.

In order to compare salts with different stoichiometry, it is better to use an expression of supersaturation weighted by the number of ions in the chemical formula of the compound, n,

(16) $$S={\left(\frac{I\,A\,P}{K_{s\,p}}\right)}^{1/\nu }$$

G, Gibbs function; V, volume; P, pressure; S (Niskanen et al., 1994), entropy; T, temperature; μ, chemical potential; n, number of moles; R, gas constant; a, activity coefficient; aq, aqueous; Ksp, solubility Product; IAP, ionic activity product; S (McCrindle et al., 2017; Seethapathy and Noureddine, 2019), supersaturation.

**BOX 2. Activity coefficient and ionic strength, *I.***

The ionic strength, *I*, is the measure of the total amount of all the ions present in solution:

$$I=0.5\,\sum c_{i}\,z_{i}$$

where *z*_*i*_ is the charge of ion *i*. The main ionic components of human serum are listed in Table 1. Applying this equation, the resulting ionic strength would be: *I* = 0.141 M. However, in the following we will consider a value of 0.15 M for the ionic strength in blood, because this value is commonly accepted and generally used in the manufacturing of the blood replacement media (Nel et al., 2009).

Among the several expressions for calculating the activity coefficient based on the ionic strength the most commonly accepted one is the Debye–Hückel expression, as proposed by Davies:^6^

$$l\,o\,g\,\gamma_{i}=-A\,z_{i}^{2}\,\left(\frac{\sqrt{I}}{1+\sqrt{I}}-0.3\,I\right)$$

where A is 0.52, at 37°C.^7^ At an ionic strength of *I* = 0.15, for Ca^2+^ and HPO_4_^2–^ ions, γ = 0.33, and for PO_4_^3–^, γ = 0.08.

**TABLE 1:** Molar concentrations of ionic components in human serum with a concentration above 0.1 mM.

	Conc. (mmol/L)			
Element	Min.	Max.	Mean	References
Na^+^	135	148	141.5	Harrington et al., 2014
K^+^	0.4	5.5	2.9	Harrington et al., 2014
Ca^2+^	2.3	2.6	2.5	Harrington et al., 2014
Mg^2+^	0.7	0.9	0.8	Harrington et al., 2014
Cl^–^	95	105	100	Reid et al., 2003
HCO_3_^–^	24	32	28	Reid et al., 2003
Pi	1.12	1.45	1.3	Aduen et al., 1994
C_3_H_5_O_3_^–^			1.1	Oddoze et al., 2012

For a given ion concentration, **ion activity decreases with the ionic strength**. For example, for *I* = 0.15, the activity of the Ca^2+^ ion is reduced threefold with respect to the Ca^2+^ concentration, and the activity of the PO_4_^3–^ ion decreases 10-fold. Such reductions ultimately prevent spontaneous crystallization in blood and, thus also, for example, invalidate the hypothesis that CKD- or ESRD-related hyperphosphatemia *per se* causes precipitations seen in MVC.

### Calcium Phosphate Precipitation System

Calcium ions in aqueous solution are not totally free rather they are solvated with water (Zavitsas, 2005). In other words, they form a coordination sphere of water molecules. This coordination sphere is composed of an inner shell of six water molecules with strong binding energy and an outer shell of 0–6 water molecules with weaker binding energy. The strong hydration of calcium is an important factor in the crystallization of calcium salts from water. Actually, the detachment of water molecules from calcium ions occurring on a crystal surface before they are incorporated into the crystal lattice is often the rate-limiting factor in crystal growth kinetics (van der Voort and Hartman, 1991). This is the case, for example, in calcium oxalate crystallization, for which trihydrate and dihydrate salts are kinetically favored and precipitate earlier than the monohydrate polymorph that has lower solubility and higher thermodynamic stability (Grases et al., 1990).

There are three different phosphate ions derived from the dissociation of phosphoric acid (H_3_PO_4_): H_2_PO_4_^–^, HPO_4_^2–^, and PO_4_^3–^. In aqueous solution produces several species of phosphate ions according to the following equilibria (Vega et al., 1994):

$$H_{3}{\mathrm{PO}}_{4\,\left(s\right)}+H_{2}O_{\left(l\right)}\Leftrightarrow H_{3}O_{\left(\mathrm{aq}\right)}^{+}+H_{2}{\mathrm{PO}}_{4}^{-}{}_{\left(\mathrm{aq}\right)}$$

$$K_{\mathrm{a1}}\,\left(37^{\circ }\,C\right)=0.0170$$

H2PO4-+(aq)H2O(l)⇔H3O++(aq)HPO42-(aq)

$$K_{\mathrm{a2}}\,\left(37^{\circ }\,C\right)=7.7569\times 10^{-7}$$

HPO42-+(aq)H2O(l)⇔H3O++(aq)PO43-(aq)

$$K_{\mathrm{a3}}\,\left(37^{\circ }\,C\right)=4.2180\times 10^{-13}$$

**FIGURE 1:**
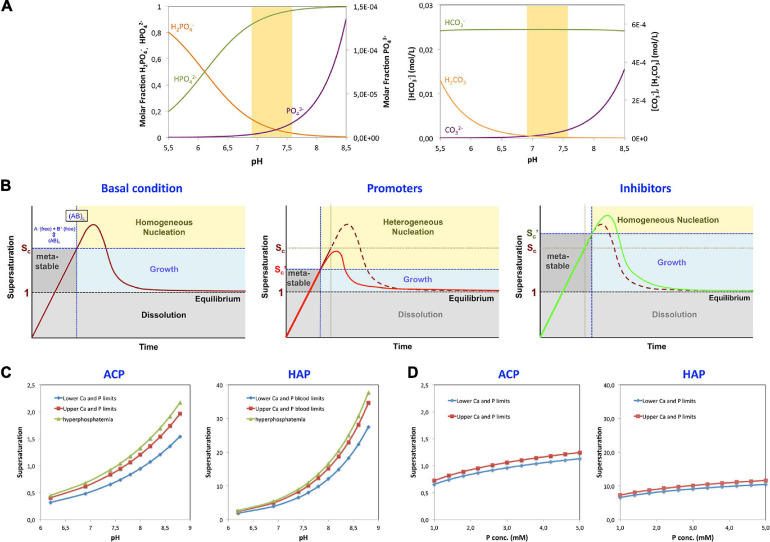
Parameters affecting precipitation. **(A)** La Mer plots representing the different stages of precipitation from solution in relation to supersaturation under different conditions are shown. Initial conditions (left panel): the supersaturation limit value (Virchow, 1858) and a metastable range of ion activities in which the solution does not precipitate, despite being supersaturated, are depicted. This metastable region represents an energy barrier for nucleation. Once a critical concentration is reached (Sc), homogeneous precipitation will take place in the solution. The limits can be reduced in the presence of nucleation promoters (central panel). Promoters in solution have a high affinity for any of the crystal component ions and can induce nucleation even below the critical supersaturation level through a process called heterogeneous nucleation. On the other hand, other molecules, termed inhibitors of nucleation, have the capacity to increase the energy barrier for nucleation, thereby increasing both the critical supersaturation value and the induction time for nucleation (right panel). **(B)** Effect of pH on the formation of the different species of phosphate and carbonate. Left, molar ratio of the phosphate species H_2_PO_4_^–^ (orange), HPO_4_^2–^ (green), and PO_4_^3–^ (violet) in solution as a function of pH, within the pH range of interest: 5.5–8.5. Right, variation of the concentrations of H_2_CO_3_ (orange), HCO_3_^–^ (green), and CO_3_^2–^ (violet) within the same pH range in DMEM medium. The shaded area corresponds to the expected physiological pH. **(C)** Effect of pH on the supersaturation of ACP and HAP in blood at the lowest and highest concentrations of Ca and phosphate (Pi), at normal concentration limits and under hyperphosphatemic conditions. **(D)** Effect of the total concentration of phosphate (Pi) on the supersaturation of ACP and HAP in blood, at pH = 7.4. The lower limit corresponds to the Ca and Pi concentrations of 1.02 and 1.00 mM, respectively; the upper limit corresponds to 1.23 and 1.5 mM; and hyperphosphatemia corresponds to 1.23 and 2.00 mM.

Their relative presence in a medium depends on the pH (Figure 1A). At the physiological pH = 7.4, the concentrations of H_2_PO_4_^–^, HPO_4_^2–^, and PO_4_^3–^ are 0.049, 0.95, and 1.08 × 10^–5^, respectively. In solutions with a pH of 6.8, the concentrations change to 0.17, 0.83, and 0.27 × 10^–5^, respectively. In solutions with pH of 7.6, the concentrations become 0.031, 0.97, and 1.71 × 10^–5^. Thus, in this pH – range of 6.8–7.6, the variations of HPO_4_^2–^ are minimal, but the concentration of H_2_PO_4_^–^ is reduced more than fivefold and that of PO_4_^3–^ is increased in a similar proportion (see Box 3).

**BOX 3. Dissociation constants of calcium and phosphate ions.**

The amount of free calcium and phosphate ions in solution available for precipitation is reduced by the formation of soluble calcium phosphate. The equilibrium equations and corresponding association constants of these complexes are as follows (Chughtai et al., 1968):

${\text{Ca}}^{2+}+{\text{H}}_{2}\,{\text{PO}}_{4}^{-}⟺{\text{CaH}}_{2}\,{\text{PO}}_{4}^{+}\;$*K* = 31.9 l/mol

${\text{Ca}}^{2+}+{\text{HPO}}_{4}^{2-}⟺{\text{CaHPO}}_{4}\left(\mathrm{aq}.\right)$     *K* = 6.81 × 10^2^ l/mol

${\text{Ca}}^{2+}+{\text{PO}}_{4}^{3-}⟺{\text{CaPO}}_{4}^{-}$      K = 3.46 × 10^6^ l/mol

The different phosphate ions can combine with calcium ions into 12 different compounds, although only six can be produced directly from solution. The empirical formulas and solubility products of these six compounds are given in Table 2.

**TABLE 2:** Main calcium phosphate compounds appearing in precipitates from aqueous solutions.

Compound, acronym, formula (mineral)	Ionic Product	K_*sp*,_ 37°C	pH	References
Monocalcium phosphate monohydrate, MCPN, Ca(H_2_PO_4_)_2_⋅H_2_O		soluble^(c)^	<2	Madsen and Thorvardarson, 1984
Dicalcium phosphate, DCP, CaHPO_4_ (Monetite)	aCa×2+aH⁢P⁢O43-	2.51 × 10^–7^	<4	Madsen and Thorvardarson, 1984; Tamimi et al., 2012; Shi et al., 2017
Dicalcium phosphate dihydrate, DCPD, CaHPO_4_⋅2H_2_O (Brushite)	aCa×2+aH⁢P⁢O43-	2.19 × 10^–7^	3.5–6.8	Moreno et al., 1966
Octocalcium phosphate, OCP, Ca_8_H_2_(PO_4_)_6_⋅5H_2_O	$a_{C\,a^{2+}}^{4}\times a_{H^{+}}\times a_{H\,P\,O_{4}^{3-}}^{3}$	2.01 × 10^–49^	∼6	Tung et al., 1988
**Hydroxyapatite, HAP, Ca_9_(PO_4_)_6_(OH)_2_**	${\text{a}}_{{\mathrm{Ca}}^{2+}}^{10}\times {\text{a}}_{{\mathrm{HPO}}_{4}^{3-}}^{6}$$\times {\text{a}}_{{\mathrm{OH}}^{-}}^{2}$	**5.5** × **10**^–^**^118^**	**9.5–12**	McDowell et al., 1977
**Amorphous calcium phosphate, ACP2^(a)^, CaH_0.22_(PO_4_)_0.74_⋅nH_2_O^(b)^**	**a**_**Ca**_^**2 +**^ $\times {\text{a}}_{H^{+}}^{0.22}$$\times {\text{a}}_{{\mathrm{PO}}_{4}^{3-}}^{0.74}$	**3.35** × **10**^–**12**^	**7.4**	Christoffersen et al., 1990

*^(a)^Two types of ACP have been described, here ACP2 with Ca/P ratio of 1.35 is considered.*

*^(b)^*n* = 3–4.5; 15–20% H_2_O.*

*^(c)^Solubility, *S* = 783.1 g/L. Bold face – species relevant in human pathobiology.*

The important question is which of those species may be relevant in MVC? An analysis of *in vitro* and *in vivo* calcifications shows that from the six different calcium phosphate solid compounds (Bohner, 2010), only amorphous calcium phosphate (ACP) and hydroxyapatite (HAP) are generally present in MVC (Chernov, 1984; Mullin, 2001; Klaus-Werner, 2019).

ACP is usually the first phase to precipitate under physiological conditions (Manas et al., 2012; Hortells et al., 2017; Lotsari et al., 2018), and therefore it is the relevant early phase in pathophysiology of ectopic calcifications. Importantly, the ACP and HAP are mainly composed of PO_4_^3–^ ions as confirmed by IR analysis (Vecstaudza et al., 2019). Therefore, although the concentrations of H_2_PO_4_^–^ and HPO_4_^2–^ ions are substantially higher than that of PO_4_^3–^ ions at physiological systemic pH values, it appears that in MVC the concentration of **PO_4_^3^**^–^ **ions** will be the most critical one.

Given that ACP is the first compound to be observed in the MVC process (Hortells et al., 2017), the supersaturation of this compound (*S*_*ACP*_) within the media will likely predict and trigger the appearance of MVC. To calculate *S*_*ACP*_, reliable values of K_*sp*_(ACP) in biological tissues would be needed. However, the only value of Ksp available at present K_*sp*_(37°C) = $a_{C\,a^{2+}}\times a_{H^{+}}^{0.22}\times a_{P\,O_{4}^{3-}}^{0.74}$ = 3.35 × 10^–12^ was determined *in vitro*, while allowing for a wide variation of pH values (Christoffersen et al., 1990). Such pH variation is unlikely occur in the living tissues closely controlling the pH homeostasis. Therefore, the Ksp(ACP) would need to be determined to reflect the conditions within the media. Meanwhile, we have used the *in vitro* K_*sp*_ (ACP) value in our calculations of ACP supersaturation keeping in mind that the results can be only approximates. The results are provided and compared with those of dicalcium phosphate dihydrate (DCPD), octacalcium phosphate (OCP), and HAP in normal subjects in Table 3. These calculations show that within the biological range of minimum and maximum calcium and phosphate concentrations in humans, the supersaturation should vary from *S* = 0.65 to *S* = 0.83. During the hyperphosphatemic conditions in patients with CKD, *S* equals 0.92, which is still below the precipitation level (*S* ≥ 1). However, the local changes in pH within the media or imbalance between the promoters and inhibitors could favor precipitation, as shown below. The changes in local pH within the media could explain the fact that MVC appears in CKD before hyperphosphatemia is observed (Hortells et al., 2017), may be as a consequence of VSMC transdifferentiation, and possibly also the occurrence of MVC observed in non-CKD conditions, such as diabetes or aging.

**TABLE 3:** Supersaturation, *S*, of the various CaP species (ACP, DCPD, OCP, HAP) at several experimental conditions, at 37 °C.

[Ca^2+^]_*o*_ mM	[PO_4_^3–^]_*o*_ mM	Medium	[HCO_3_] mM *pH_0_-pH_∞_*	*S* (ACP2)	*S* (DCPD)	*S* (OCP)	*S* (HAP)
1.02	**1**	Blood (min.)	7.40	0.65	0.32	3	7
1.23	**1.5**	Blood (max.)	7.40	0.83	0.41	4	8
1.23	**2**	Hyperphosphatemic	7.40	0.92	0.48	5	9

*Minimal and maximal values of calcium and phosphate in blood of healthy subjects (Larsson and Ohman, 1978; Boron and Boulpaep, 2009).*

### Molecular Processes in CaP Precipitation

The knowledge of the molecular mechanisms of the physiological bone and teeth mineralization may be helpful to understand ectopic calcifications, as they may share common mechanisms (Allen and Moe, 2020). In summary, physiological calcification is a complex phenomenon carried out by specialized cells, and it involves a variety of actors, mainly proteins that in the bone constitute the osteoid: (a) collagenous proteins and elastin that, in the vascular wall, create close compartments to facilitate confined crystallization and crystal growth (Veis, 2005); (b) amphiphilic proteins self-assembled to form scaffolds to provide potential nucleation sites before mineralization begins (Beniash et al., 2000); and (c) non-collagenous proteins that promote nucleation and control crystal growth morphology through interactions with certain crystal faces (Veis, 2005). In the first stage of biomineralization, an amorphous inorganic compound is produced in an environment occupied by a majority of organic compounds (60% of the total volume; 82). In the second stage, the inorganic compound crystallizes, and the crystals are organized into highly hierarchical superstructures. This complex biological machinery exercises complete control over the crystallization process, including crystal size, crystalline purity, growth orientation, shape molding, and hierarchical superstructuring.

**FIGURE 2:**
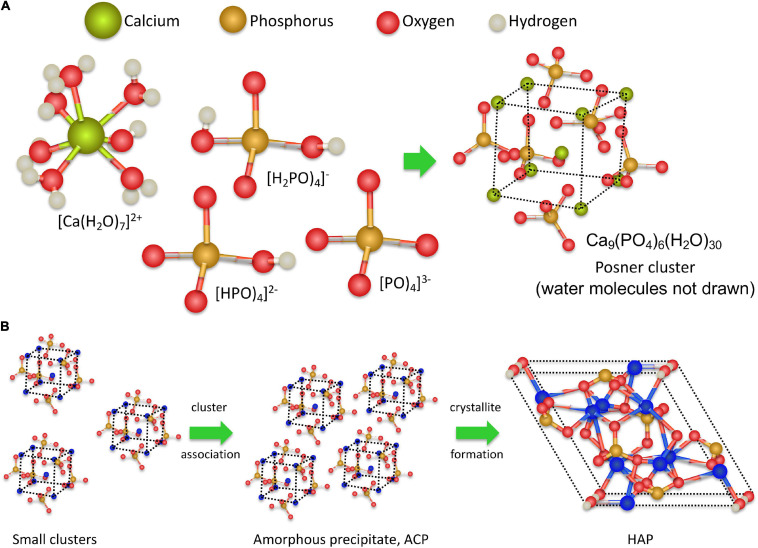
Promoters of CaP mineralization. **(A)** Structure of major biomolecules that as promoters of calcification: phosphorylated proteins, sulfated glycosaminoglycans, carboxyglutamic proteins and phospholipid membranes. **(B)** CaP mineralization in the presence of nucleation promoters: accumulation of Ca by absorption on promoters, precipitation of ACP, and crystallization of HAP.

**FIGURE 3:**
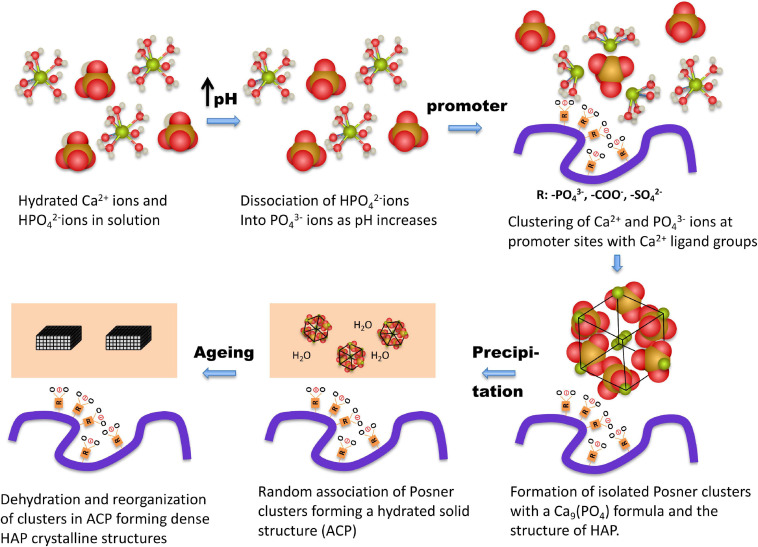
Hypothetical molecular processes in calcium phosphate medial vascular mineralization. At pH up to 7.40 Ca^2+^ ions are hydrated, and the majority of the phosphate molecules are in the form of HPO4-^2^- ions. The precipitation is triggered by a local increase of pH to above 7.90, associated with a marked increase in PO_4_^3–^ ions; the main component in ACP and HAP. Ca^2+^ and PO_4_^3–^ ions form complexes of increasing coordination number, eventually forming multinuclear clusters, which contain nine Ca^2+^ ions and six PO_4_^3–^ ions. Subsequently, promoter macromolecules with charged Ca^2+^-ligand groups (phosphate, carboxylate, and/or sulfate) produce a local accumulation of Posner clusters and the appearance of ACP precipitates. Finally, as the process progresses, the precipitate clusters rearrange into dense crystalline HAP nanoparticles.

Recent analyses of the early stages of CaP precipitation using high-resolution molecular techniques and *ab initio* molecular dynamics simulations (Lin and Chiu, 2018; Zhao et al., 2018) provide better insights into the molecular processes involved in CaP precipitation (Habraken et al., 2016). During the initial step, calcium and phosphate ions associate to form dinuclear and trinuclear complexes and subsequently polynuclear Posner clusters (Beniash et al., 2000) composed of nine Ca^2+^ ions and six phosphate PO_4_^3–^ anions, surrounded by 30 water molecules, as depicted in Figures 2A, 3. After that, Posner clusters agglomerate to form hydrated precipitates with a low density and a Ca/P ratio of 1:1 (Zhang et al., 2015; Jiao et al., 2016; Niu et al., 2017). In the third step, the precipitate becomes denser by losing water and increasing the Ca-O-P connectivity to form ACP, with a Ca/P ratio of 1:1.35. Finally, through slow transformation in the solid state, crystalline HAP nanoparticles are formed within the precipitate (Figure 3). This mechanism does not follow classical nucleation theory that is based on the one by one incorporation of add-atoms to the growing nuclei. This also applies to the process of CaCO_3_ nucleation (Smeetsa et al., 2017). One of the consequences features of this non-classical behavior is the lowering of the nucleation energy barrier (see below) (Yang et al., 2019).

Amorphous calcium phosphate precipitates gradually transform into HAP (Hortells et al., 2015). The amorphous ACP solid evolves by slow reorganization of the loosely packed clusters into crystalline, rod-like domains of nanometer size, with a HAP structure (Figure 2). The appearance of HAP implies a dramatic change of the calcification regime. Because HAP is virtually insoluble, the crystallization process turns largely irreversible. It should be noted that the blood and fluids contained in biological tissues are highly supersaturated in HAP. The fact that HAP does not precipitate spontaneously in tissues appears to be due to a high nucleation barrier of this calcium phosphate phase keeping the medium in a metastable state. Once HAP has been formed, there is no energy barrier to stop growth, provided *S* > 1, unless inhibitors are present on site. Thus, in this sense at pH of 7.40 and at the lowest level of blood Ca concentration (1.02 mM), calcifications composed of HAP would be allowed to grow even when the concentration of Pi would be 1,000 times less than the lowest value in blood (see Figures 1C,D).

Transmission Electron Microscopy analyses of CaP calcifications in cell cultures are consistent with the precipitation mechanism described above (Hortells et al., 2015). Fresh precipitates appear as low-density sheets having a Ca/P ratio of one and showing some areas of higher electron density. After some aging time, precipitates show spherulite formations, which do not exhibit any crystalline order in electron diffraction and have a Ca/P ratio of 1.35, like ACP. After longer aging periods, precipitates show rod-like nanoparticles that yield electron diffraction rings, and they show crystal planes at high resolution. Based on observations of in a rat model using scanning electron microscopy (SEM), the arterial calcifications are localized and not uniformly distributed, suggesting a heterogeneous nucleation induced by a promoter, or a local increase in supersaturation, or both mechanisms as the origin of the calcification (Hortells et al., 2017). A chemical analysis of the deposits yielded mostly a Ca/P ratio of 1.35, close to that of ACP, although occasionally higher values were found, approaching that of HAP (1.67). Rat arteries that did not show any sign of calcification in optical microscope, at closer observation by SEM revealed small areas with a strong presence of Ca, but no detectable phosphate (Hortells et al., 2017). Although this preliminary observation should be studied in greater detail, it appears possible that these Ca aggregates may actually trigger CaP deposition in similar fashion as observed in physiological calcification in bones and/or teeth with calcium-rich proteins serving as promoters of mineralization.

### Nucleation and the Activation Barrier

It is important to note that solubility products are calculated based on the activities of ions in solution that are in direct contact with the solid phase. However, in the absence of the solid phase, precipitation does not occur immediately after the ionic product exceeds the solubility product. In other words, *S* > 1 is a necessary but not sufficient condition for precipitation because there is an energy barrier for nucleation, similar to the majority of chemical reactions (Vallence, 2017) that has to be overcome before precipitation takes place. The onset of nucleation occurs when the size of the nuclei reaches a certain critical value, due to the large surface energy of small nuclei (Chernov, 1984; Mullin, 2001; Klaus-Werner, 2019). Below that critical size, the process of ion association is transient and reversible. Below that critical size, the process of ion association is transient and reversible. This is due to the fact that ions on the particle surface have greater energy than those in the bulk, given that the number of nearest neighbors of ions on the surface is obviously lower than that of ions in the bulk. The total Gibbs free energy of a particle growing from its component free ions is the sum of that of bulk ions and surface ions, and for small sizes, the number of surface ions is relatively large, so they tend to re-dissolve until the supersaturation in solution reaches the critical value. The process of formation of stable nuclei requires a definite induction time defined by the period between the time the critical supersaturation has been established and the first nuclei were detected (Chernov, 1984; Mullin, 2001; Klaus-Werner, 2019). As the size of the nuclei increases, they can grow with decreasing levels of supersaturation. Finally, large crystals will continue to grow even at very low levels of supersaturation, as shown in Figure 2. Thus, while induction of nucleation requires high levels of supersaturation, once larger particles have been formed, the growth will proceed with ever decreasing levels of supersaturation.

Consequently, a supersaturated solution can remain in a metastable state until the activation barrier has been overcome, i.e., the critical supersaturation value, *S*_*c*_, has been reached, therefore enabling the formation of stable nuclei (Christoffersen et al., 1990; Figure 1B). Thus, ***S* (ACP) > 1 is the necessary condition for growth, and S (ACP) > Sc is the necessary condition to trigger precipitation**. Using data from spontaneous nucleation experiments in water (Strates et al., 1957; Fleish and Neuman, 1961), we have estimated the *S*_*c*_ values of 1.03 (Fleish and Neuman, 1961) and 1.05 (Strates et al., 1957) for ACP. These values represent early estimates and require validation using well-controlled experimental models emulating *in vivo* conditions. Nevertheless, these early Sc (ACP) estimates indicate that the critical supersaturation for MVC should be rather low, underscoring the non-classical character of ACP nucleation (Smeetsa et al., 2017; Yang et al., 2019). Thus indeed, the molecular mechanism of ACP nucleation appears to proceed through the gradual formation of soluble CaP complexes of increasing size, eventually forming multinuclear clusters with the formula, Ca_9_(PO_4_)_6_⋅nH_2_O (Mancardi et al., 2016). These clusters having the same structure as HAP are the likely precursors of ACP. initially forming the low-density amorphous precipitates with a Ca/P ratio of approximately 1 (ACP1) and eventually evolving into denser particles (ACP2) with a Ca/P ratio of 1.35, typically found in MVC, in bones, and other physiological and ectopic calcifications.

### Nucleation Promoters

The process of CaP precipitation described above can be altered by the presence of promoters and/or inhibitors in the system. **Promoters** are agents that favor the precipitation of compounds in solutions that otherwise would remain metastable. Conversely, **nucleation** **inhibitors** restrain the formation of precipitates from solutions that otherwise would experience spontaneous homogeneous nucleation. In thermodynamic terms, promoters decrease *S*_*c*_ and induce **heterogeneous nucleation** located at the promoter site, whereas inhibitors increase *S*_*c*_ and retard the calcification process (Figure 1B). Thus, promoters and inhibitors may control the spontaneous crystallization processes and appear to dominate MVC.

Calcification promoters typically harbor abundant Ca^2+^-ligand groups in their chemical structure, responsible for the local accumulation of these ions. In this calcium-rich environment created by the promoter, nucleation might occur with minor increases in phosphate ion activities; calcification mechanism responsible for the formation of bones and teeth (Addadi and Weiner, 1992; Beniash et al., 2000; Veis, 2005). However, the disordered texture of the deposits observed in MVC suggests that the mechanism of mineralization and the agents involved in the process are likely different. Yet the two processes, bone formation and MVC, may have in common that the **nucleation promoters can induce the onset of precipitation, because in both cases, the occurrence of deposits is discrete and localized**, both being typical features of heterogeneous nucleations. Disseminated foci of calcifications within the medial layers are the outstanding hallmark of the MVC. However, the nature of promoters in MVC has not yet been studied in detail and requires further clarification.

**FIGURE 4:**
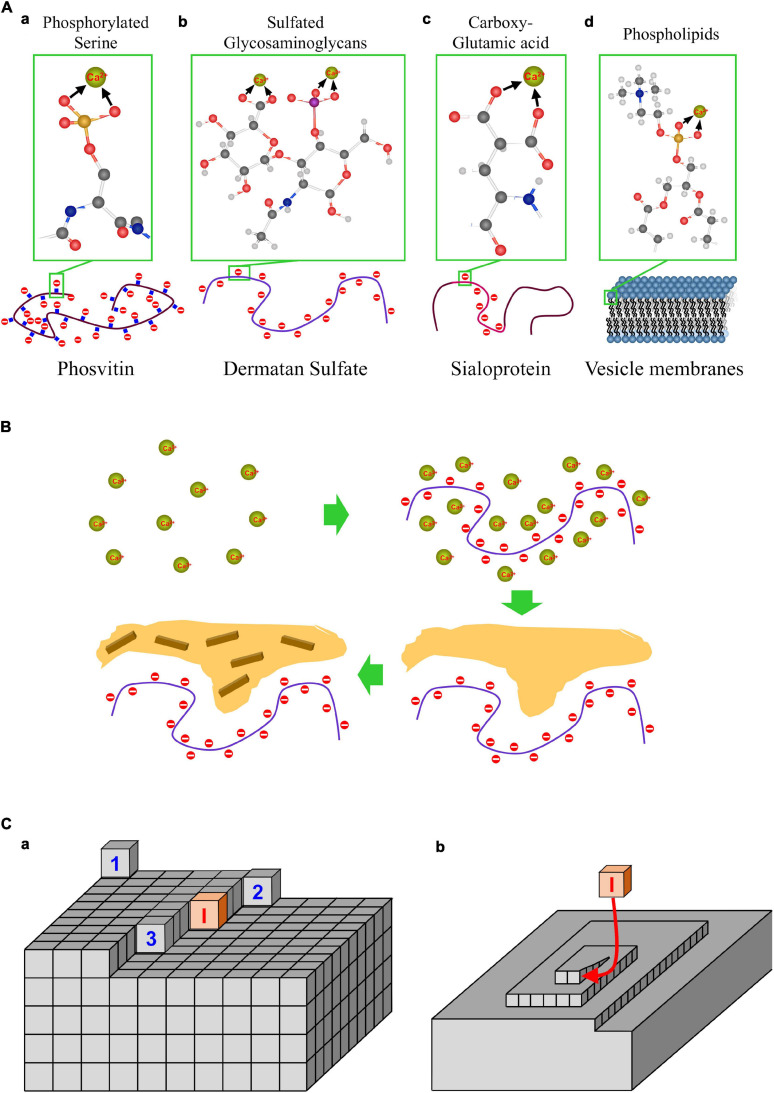
Promoters and inhibitors of CaP mineralization. **(A)** Structure of major biomolecules that as promoters of calcification: phosphorylated proteins, sulfated glycosaminoglycans, carboxyglutamic proteins and phospholipid membranes. **(B)** CaP mineralization in the presence of nucleation promoters: accumulation of Ca by absorption on promoters, precipitation of ACP, and crystallization of HAP. **(C)** Mechanism of crystal growth inhibition. **(a)** Representation of a Kossel crystal, with the different positions of adatoms on the crystal surface: flat site (surface nucleation) (Virchow, 1858), step site (Niskanen et al., 1994), kink site (Lehto et al., 1996), inhibitor molecule blocking a kink site (I); **(b)** a screw dislocation, and blocking of face growth by an inhibitor molecule.

CaP nucleation promoters are molecules or macromolecules with a high affinity for calcium and/or phosphate ions. To interact with phosphate ions, promoters must have positive charges, which in biomolecules are mostly provided by ammonium ions or quaternary amines. In any case, however, the interactions are weak. Figure 4A shows the typical Ca^2+^-ligand groups in biomolecules. In descending order according to their binding strength, these groups include phosphonate, phosphate > carboxylate > sulfonate = sulfate > alkoxide ≈ water (Zhao et al., 2018). Several kinds of biomolecules may contain large numbers of these groups, as it is the case of phosphate in **multiphosphorylated proteins** (Figure 4Aa), such as phosvitin (Greengard et al., 1964), involved in physiological calcifications (Sarem et al., 2017). Phosvitin consists of approximately 50% serine residues, which carry a phosphate group (Greengard et al., 1964) that gives this protein an extraordinary capacity to accumulate Ca^2+^ ions. Sarem et al. (2017) were able to show that the precipitation of CaP proceeded by the incorporation of Ca^2+^ ions into phosvitin, followed by the precipitation of ACP, and subsequent recrystallization into HAP by aging. The experimental conditions included HEPES buffer (pH = 8), 1.25 mM Ca(NO_3_)_2_, and 1.5 mM (NH_4_)_3_PO_4_. Under these conditions, the supersaturations of ACP and HAP correspond to 1.21 and 8, respectively. Furthermore, recently, it has been shown that the disordered secondary structure of phosvitin orchestrates the nucleation and growth of biomimetic bone such as apatite (Sarem et al., 2017).

**Phospholipids** (Figure 4Ad), the main component of cell membranes, matrix vesicles and exosomes, have also been proposed as promoters of pathogenic calcifications (Felix and Fleisch, 1976; Boskey, 1978; Schoen et al., 1988; Anderson, 2007; Wuthier and Lipscomb, 2011). Phospholipids are arranged in layers with a high density of phosphate-charged groups endowed with the capacity to concentrate Ca^2+^ ions. Indirect evidence of their role as promoters has been found in analyses of medial sclerosis of large arteries, aortic valves, and atherosclerotic plaques (Felix and Fleisch, 1976; Boskey, 1978; Schoen et al., 1988; Anderson, 2007; Wuthier and Lipscomb, 2011). Phospholipids may be released following degradation of cells or matrix vesicles (Felix and Fleisch, 1976). In addition, their phosphate groups may be also released as PO_4_^3–^ ions by alkaline phosphatase, potentially also facilitating calcium precipitation (Towler, 2005).

Promoter biomolecules containing sulfate include glycosaminoglycans (**GAGs**) (Figure 4Ab) or mucopolysaccharides (Kepa et al., 2015), such as chondroitin sulfate, dermatan sulfate, keratan sulfate, hyaluronic acid, and heparin. In combination with proteins, form mucoproteins are formed. Mucopolysaccharides and mucoproteins have been the object of intense research based on their role as a matrix for the nucleation and structuring of calcium oxalate renal calculi. Among the mucopolysaccharides, dermatan appears the most interesting one, because its presence in the skin, blood vessels, and heart valves.

Biomolecules with a large number of carboxylic residues include **glutamic- and carboxy-glutamic**-**rich proteins** (Figure 4Ac). Collagen and elastin neutral proteins have also been proposed as promoters of VC (Urry, 1971). This hypothesis is based on the observation that Ca^2+^ ions may interact strongly with protein carbonyl groups from glycine amino acids arranged in a helix conformation. However, it should be noted that the carbonyl groups can barely compete in Ca^2+^ binding with charged groups, such as phosphate, carboxylate, and sulfate ions, because these groups interact through strong electrostatic forces, in a bidentate (also named chelate and it means bonded by to atoms to the central atom) manner. Instead, most likely, carbonyl groups appear to bind to Ca^2+^ ions by means of hydrogen bonds with hydration water (see section “Key Concepts”). Therefore, glycoproteins such as bone acidic glycoprotein-75, or bone phosphoproteins such as osteopontin or bone sialoprotein (Chen et al., 1992), appear to be more suitable promoters than collagen and elastin. These proteins not only have a higher capacity to bind Ca^2+^, but they interact with collagen itself in the presence of Ca^2+^ in a concentration-dependent manner. This Ca^2+^-mediated interaction with collagen has also been observed in matrix vesicles (Kirsch and von der Mark, 1991). Furthermore, studies on Ca^2+^-collagen interactions have shown that they take place through electrostatic interactions with the carboxylate groups (Glu and Asp) present in collagen (Pang et al., 2017).

All of these biomolecules have demonstrated the capacity to promote CaP nucleation in metastable solutions *in vitro* and appear to be suitable potential candidates for triggering and sustaining the calcification process in MVC, whether they are present in exosomes, apoptotic bodies, long-life proteins or transdifferentiated cells. A scheme of CaP deposition induced by promoters is shown in Figure 4C. In the first step the promoter expedites an accumulation of Ca^2+^ ions that are trapped by the high density of calcium ligand groups in the promoter structure. In the presence of phosphate, Ca^2+^ ions will form soluble CaP clusters, eventually ending up as precipitates, as described above.

### Inhibition of Calcium Phosphate Nucleation and Crystal Growth

In addition to the promoters the onset and propagation of VC is also determined by the presence of agents that retard or restrain CaP precipitation. These agents comprise the class of inhibitors. Based on the mechanisms of action, four main groups of agents may be distinguished. The first group consists of agents precluding or retarding the formation of stable nuclei by increasing the activation barrier (nucleation inhibitors) (Giocondi et al., 2010). The second consists of agents that are in small amounts capable of restraining the crystals’ growth by attaching to the crystals’ surfaces (crystal growth inhibitors) (Dobberschuütz et al., 2018). The third constitute agents slowing the maturation from ACP to HAP and the fourth compounds that, without being truly inhibitors, can reduce supersaturation by sequestering calcium ions in the form of soluble complexes (Reznikov et al., 2016) or, in the case of renal disease, reduce hyperphosphatemia with the use of phosphate binders (Floege, 2016) or renal Pi reabsorption (Tsuboi et al., 2020), thus reducing the risk of calcification.

#### Nucleation Inhibition

Albeit the precise value of critical supersaturation required for the nucleation of ACP *in vivo* is not known and data on nucleation inhibition are sparse (Strates et al., 1957; Fleish and Neuman, 1961) approximate value can be calculated based on *in vitro* data available in the literature (Strates et al., 1957). Accordingly, the critical supersaturation for the nucleation of ACP would be *S_*c*_* = 1.05 at pH = 7.4. Interestingly, analogous calculations based on experimental data of other authors (Fleish and Neuman, 1961) yields *S_*c*_* = 1.03 at pH = 7.4. According to this value, and considering the average concentration of Ca^2+^ = 1.18 mM found in healthy subjects, the expected concentration of Pi needed for spontaneous calcification in blood would be 2.75 mM; a value far above the regular blood levels (0.97–1.45 mM) blood levels, or even of patients with severe hyperphosphatemia.

It has been suggested that active collagen may act as a promoter of calcification (Strates et al., 1957; Fleish and Neuman, 1961). However, this proposition appears rather unlikely because the calculated *S_*c*_* = 0.73 based on the solubility product of ACP reported by Christoffersen et al. (1990), would not fulfill the conditions required for precipitation *S* ≥ 1. These discrepancies emphasize the need for precise calculations of thermodynamic parameters based on well-controlled experimental studies *in vivo*. Nevertheless, Fleish’s experiment has also demonstrated the nucleation inhibition capacity of pyrophosphate (PPi) and polyphosphate by increasing the *S_*c*_(ACP)* for spontaneous (homogeneous) precipitation up to 1.07 and 1.10, respectively. Apparently, the critical supersaturation values for nucleation are rather low, but this conclusion should be considered with caution, given that both experiments—as well as the determination of *K*_*sp*_(ACP)—were performed at different ionic strengths and under changing pH conditions (pH has a huge effect on CaP precipitation). Actually, the *K*_*sp*_ was calculated at *I* = 0.035, and the pH changed from 7.4 to 5.7 during precipitation; while in Strates et al. (1957) the ionic strength was 0.165 and the final pH was around 5.9. Nevertheless, despite of the obvious shortcomings of calculations of thermodynamic parameters based on *in vitro* data, these calculations offer an important impetus to explore further the role of collagen in CaP nucleation promotion by studying the relevant thermodynamic parameters *in vivo.* Furthermore, they also appear to explain the ability of pyrophosphate and organic polyphosphates to inhibit or to retard the onset of CaP nucleation. Strates et al. (1957) also described that HAP seed crystals can grow in normal blood serum, a proposal that supports our hypothesis that the nucleation of CaP deposits is controlled by ACP precipitation kinetics, but once the transformation to HAP occurs, the kinetics are driven by HAP crystal growth, which will progress even when the alterations that provoked the mineralization event return to normal. Actually, the threshold of P_*i*_ concentration for HAP supersaturation at pH = 7.40 and at the lower limit of the total calcium concentration in blood (1.01 mM) could be as low as 0.85 mM.

#### Inhibitors of ACP Conversion Into HAP

Some molecules are capable of stabilizing ACP and preventing or retarding the maturation into HAP, thereby keeping the door open to reversion of mineralization, which would become extremely improbable once HAP has been formed. Two known inhibitors of calcification acting by the ACP stabilization are pyrophosphate (PPi) (Ibsen and Birkedal, 2018) and magnesium (Boskey and Posner, 1974; Ter Braake et al., 2018). For details, see Box 4.

**BOX 4. Crystal growth inhibition.**

Inhibitors of crystal growth are molecules or ions that attach firmly to the crystal surface, thereby making it difficult for the growing crystal units to displace them thus preventing or retarding the attachment of new adatoms. The effect of inhibitors on crystal growth is briefly explained. If the ions in a solution remain above the nucleation rate, then the supersaturation will remain above the critical value for nucleation, Sc, allowing the process to go on. Initially, crystals have a rounded shape and a rough surface, but gradually they develop flat faces to minimize their surface energy. Using the model of a Kossel crystal (Kossel, 1927; Stranski, 1928; Figure 4C) with cubic adatoms that bind to the nearest neighbors on each crystal face, the energy gain of an adatom incorporating on a completely flat face will be obviously low (Figure 4Ca, adatom 1). Yet once attached, it will create a stairs-like step facilitating the adsorption of the next adatom to the two nearest neighbors (Figure 4Ca, adatom 2). Once an adatom incorporates into a step of the stair it forms a kink site, such that the next adatom will bind to the three nearest neighbors (Figure 4Ca, adatom 3). Some inhibitor molecules may block the kink sites (Figure 4Ca, I), therefore restraining the growth of the face. This mechanism of surface nucleation and the spreading of steps to form new layers require high levels of supersaturation. However, if the face has a defect, called a screw dislocation (Figure 4Cb), permanent step site is created, and the supersaturation required for crystal growth decreases considerably. In this manner, only a small number of inhibitor molecules attached to the screw dislocations may very effectively restrain the growth of a crystal face. Inhibitors will be less effective on a face growing through a secondary nucleation mechanism. Furthermore, in the case of rough faces that consist mostly of kink sites, large amount of inhibitor molecules will be required to block the growth. It should be kept in mind that, ultimately, the type of the prevailing mechanism will depend largely on the level of supersaturation in the following order of importance: rough growth > surface nucleation > screw dislocation.

Available reports on CaP crystal growth inhibition refer mostly to HAP. The best well-known inhibitor of HAP crystallization is PPi. This ion is firmly adsorbed on a HAP crystal surface (Fleisch, 1978; Ibsen and Birkedal, 2018) affecting the progression of the mineralization process in a number ways including an increase in the critical supersaturation for both homogeneous and heterogeneous nucleation, lengthening of the nucleation induction period, change of the crystal morphology, and crystal stabilization by reduction of the growth and the dissolution rate. Although less effective, other molecules such as citrate (Mekmene et al., 2012; Shao et al., 2018) and magnesium (Boskey and Posner, 1974) can also be considered HAP crystal growth inhibitors. Thus, while PPi and Mg^2+^ appear to limit the growth of HAP deposits, they are unable to reverse them. An ambitious *in vivo* experiment of CKD in rat seemed to have suggested the ability of Mg^2+^ to revert VC (Díaz-Tocados et al., 2017), however, the experimental design appears to favor the accumulation of the reversible amorphous CaP rather than HAP crystal formation.

The list of potential inhibitors of HAP crystal growth can be further extended to phosphocitrate, polyphosphates, bisphosphonates, carboxyphosphonates, phytate (Thomas and Tilden, 1972), and other not yet identified plasma components (Rufenacht and Fleisch, 1984). However, because these *in vitro* studies on nucleation progression inhibition have been performed at pH, temperature, and concentration values far from physiological or even pathophysiological conditions, their validity for *in vivo* processes is limited (Hunter and Goldberg, 1993) yet potentially useful in specific clinical settings such as renal calciphylaxis (Millan, 1990; Perelló et al., 2018; Calciphyx, 2019).

The majority of the available reports focus on the role of PPi in the prevention of mineralization (Lomashvili et al., 2004) and the role of alkaline phosphatase (AP) responsible for the hydrolysis of the PPi into phosphate (Towler, 2005; Haarhaus et al., 2017). However, due to the significant variability of the experimental *in vitro* and *in vivo* study designs, only tentative conclusions regarding the promotion and inhibition of CaP crystallization are feasible at present. To obtain full insight into the mechanisms of calcifications, standard protocols compatible with the *in vivo* conditions within the medial layer, preferably those in encountered in humans, will be required.

### Nucleation Promoters That Act as Growth Inhibitors

In some cases, nucleation promoters may possibly also act as crystal growth inhibitors based on their capacity to bind and accumulate Ca^2+^ and to adsorb on CaP crystal surfaces. This is the case of GAGs with respect to calcium phosphate brushite (Zhai et al., 2019). Occasionally, the inhibition of crystal growth by typical nucleation promoters [i.e., chondroitin sulfate (CS) and mucoproteins] has been claimed in batch precipitation reports (Hunter and Goldberg, 1993). But this apparent inhibition effect may just be the result of Ca^2+^ absorption and the consequent decrease of the supersaturation. In constant composition studies, which more accurately reflect the conditions in blood vessels, CS has promoted precipitation. Phosvitin is a special case, which behaves like a nucleation inhibitor in dissolution but promotes nucleation when it is immobilized on a collagen surface (Onuma, 2005). Protein immobilization is also decisive in the promoter-inhibitor behavior of osteocalcin, mucoproteins, phosvitin, and phosphophoryn.

### Effect of pH on Phosphate and Carbonate Nucleation

Of the three phosphate ion species, only PO_4_^3–^ participates in all stages of precipitation, as evidenced by: (i) PO_4_^3–^ is the main component of soluble precursor clusters; (ii) PO_4_^3–^ is the main phosphate ion in the first precipitating phase, ACP; and (iii) PO_4_^3–^ is also the phosphate component of HAP. As outlined above, the concentration of PO_4_^3–^ in solution is very low at pH = 6.9, (Figure 1A), but it increases rapidly with increasing pH. Consequently, the supersaturation of ACP, and the risk of CaP precipitation, is highly dependent on the pH (Figure 1C): the pH has a much greater impact than a variation in the phosphate concentration. Actually, an increase in local pH to 7.90 would be enough to reach the critical supersaturation value (*S* = 1.03) estimated for the average blood Ca and P_*i*_ concentration in healthy subjects. In physiological Ca^2+^ and pH conditions, for example, it would be necessary to increase the local concentration of Pi to 3.0 mM to reach that *S* value, which is far above the usual level in hyperphosphatemic patients. *In vitro* experiments assessing the effect of cell activity onto the calcification process have also shown a notable importance of pH, mainly as a consequence of the used of highly bicarbonate media and low CO_2_ concentration in the atmosphere (Hortells et al., 2015). Significant changes in local pH *in vivo* in contrast to the serum’s strictly regulated pH homeostasis, could arise, as explained below, through a modification of the activities of Na^+^/H^+^- and bicarbonate exchangers and of proton pumps, carbonic anhydrases, and other factors related to VSMC’ metabolism (Leibrock et al., 2016; Yuan et al., 2019). Although local alkalinity has not been demonstrated to play a role in the pathogenesis of MVC, it could be hypothesized by combination of mechanisms, such as increased presence of promoters, depletion of inhibitors possibly orchestrated by a perfect metabolic storm signifying the transdifferentation of VSMC.

With respect to calcium carbonate, it is present in considerable amounts in pathological calcifications (Bazin et al., 2012) and in *in vitro* calcifications (our own experiments). The concentration of CO_3_^2–^ ions also increases rapidly above a pH of 7 (Figure 1A), and the supersaturation of CaCO_3_ in blood can be higher than that of ACP under physiological conditions (Table 4) supporting the hypothesis that VC may be, at least in part, related to pH rather than phosphate concentration’s changes. In culture media, MEM or DMEM (media commonly used in the *in vitro* calcification procedures) maintained at 5% CO_2_ atmosphere at given concentrations of bicarbonate and the pH (Hortells et al., 2015), the supersaturation of calcium carbonate (CaCO_3_) exceeds that of CaP in both media. This finding may not only explain the co-precipitations of CaCO3 with CaP but may also suggest its role in seeding calcium phosphate nucleation sites.

**TABLE 4:** Supersaturation of ACP and CaCO_3_ in blood and in MEM [(NaHCO_3_) = 2.2 g/L], and DMEM media with different concentrations of calcium and phosphate ions.

Medium	pH	[CO_3_]_*t*_ mM	[Ca^2+^]_*o*_ mM	[PO_4_^3+^]_*o*_ mM	S(CaCO_3_)	S(ACP2)
Blood (min.)	7.4	23	1.02	1.0	1.02	0.65
Blood (max.)	7.4	30	1.23	1.5	1.63	0.83
MEM	7.93	26.19	1.8	1	2.64	1.11
MEM	8.00	26.19	1.8	3	2.86	1.85
DMEM	8.45	44.05	1.8	1	6.30	1.66
DMEM	8.40	44.05	1.8	3	5.95	2.36

*MEM, Minimal Essential Medium; DMEM, Dulbecco’s Modified Eagle Medium. The values of CaCO_3_ supersaturations were determined according to the following equilibrium constants:*

*CO_2_ + H_2_O ⇔ H_2_CO_3_      K_*h*_ (25°C) = 5.88 × 10^2^*

*CO_2_(g) ⇔ CO_2_(aq)      1/K_*d*_ (25°C) = 29.8*

*H_2_CO_3_ ⇔ HCO_3_^–^ + H^+^      K_*a1*_ (25°C) = 2.51 × 10^–4^*

*HCO_3_^–^ ⇔ CO_3_^2–^ + H^+^      K_*a2*_ (25°C) = 4.69 × 10^–11^*

*(H_2_CO_3_ + CO_2_) ⇔ HCO_3_^–^ + H^+^      K_*a*_(app) (25°C) = 5.01 × 10^–7^*

*Ca^2+^(aq) + CO_3_^–^ (aq) ⇔ CaCO_3_(s)      K_*sp*_ (25°C) = 4.69 × 10^–11^.*

### Precipitation in Intracellular or Extracellular Matrix Environments

To date, studies in the real *in vivo* or *ex vivo* medial layer environment are not available. Thus, the hypothesis charting the pathogenesis of CaP precipitation thermodynamics in the medial layer must be based only on the currently available incomplete evidence derived largely from *in vitro* observations.

Medial layer consists of ECM comprising matrisome with a number of different glycoproteins and proteoglycans with embedded networks of collagen and elastic fibers (Hynes and Naba, 2012) and VSMC. Due to the extensive extra- and intracellular compartmentalization and due to the higher viscosity of both ECM and VSMC cytoplasm compared with the water solutions and other solvents’ the mobility of the ions will be less predictable and more restricted. However, based on the available data, the viscosity of cytoplasm is approximately 1.2–1.5 times higher compared to water (Fushimi and Verkman, 1991; Bicknese et al., 1993; Kao et al., 1993; Luby-Phelps et al., 1993; Chang H. C. et al., 2008). To our knowledge, no data on viscosity of the ECM are available. Thus, as a matter of approximation, we assume that the environment of the media corresponds to viscous solution rather than solid gel. Based on these scanty reports we tentatively conclude that an increase in viscosity as suggested in the medial layer will mainly affect the ion transport, leading to a diffusion-controlled CaP crystal growth, possibly the single most important difference to the crystal growth *in vitro* solutions.

However, it is important to understand that in any biological system regardless of the composition and physical-chemical properties such as viscosity, the laws of thermodynamics and the links between supersaturation and CaP crystals’ growth retain their validity.

It is well known that electrochemical gradient between the micromolar concentration of Ca^2+^ in the VSMC cytoplasm and the millimolar extracellular Ca^2+^ concentration can be only maintained at high energy cost. This extremely low [Ca^2+^_*i*_] in cytosol along with the presence of PPi prevent spontaneous CaP precipitation inside the cell under physiological conditions [the cytosol also contains 3–5 mM free Pi (Peacock, 2021)] and normal metabolic states. Disruption of energy supply by the mitochondria, oxidative stress, electrophilic insults, etc., will cause uncontrolled influx of [Ca^2+^_*i*_] from the extracellular space or endoplasmic reticulum followed by breakdown of intracellular homeostasis and cell death (Orrenius et al., 2013). Apoptotic bodies or cell debris could become nucleation sites and thus contribute to calcification.

The hypothesis of the medial CaP crystallization’s stepwise process outlined in the section “Molecular Processes in CaP Precipitation” has received support by experimental data from Transmission Electron Microscopy analysis of CaP deposits from cell cultures and rat arteries demonstrating that early CaP deposits consist of ACP undergoing progressive densification process and resulting in the crystallization of HAP nanoparticles (Villa-Bellosta et al., 2011; Hortells et al., 2015, 2017). The fact that the two methods of VC research, *in vitro* and *in vivo*, show similar early deposits but both calcification processes are unrelated (*in vitro* being alkali-mediated homogeneous precipitation, whereas *in vivo* is heterogeneous precipitation caused by promoter nucleation) clearly shows that deposit formation and crystal maturation thermodynamics are constant and similar, independently of the environmental setup (Hortells et al., 2015).

Given the impact of pH on the supersaturation of ACP and therefore potentially initiation of calcifications, changes in pH should be considered as a potential important factor in MVC, in both intra- and extracellular milieu. The pH in both milieus is determined by a number of factors including the metabolic production of the acid moieties, the transmembrane protons’ transport, the activity of bicarbonate transporters and exchangers, and the enzymatic synthesis of bicarbonate. While the intravasal homeostasis of pH is strictly controlled within narrow boundaries, the local tissue pH appears less stable depending on the type of the tissue and specific metabolic activities (Martin and Jain, 1994). Osteoblasts, for example, thrive *in vitro* at alkaline pH (Galow et al., 2017), in agreement with the effect of metabolic alkalosis increasing osteoblastic collagen synthesis and reducing bone resorption related to a decrease in osteoclastic beta-glucuronidase release (Bushinsky, 1996). Albeit it has never been demonstrated as yet, it is tempting to conclude that local changes in tissue pH possibly caused by contractile or partially *trans*differentiated VSMC could be involved in triggering MVC, particularly if combined with nucleation promotion and VC inhibitor depletion.

Local interstitial-intracellular pH changes are interdependent and determined by both local and systemic factors. In VSMC, several transporters participate in the movement of protons and bicarbonate to quickly control intracellular pH (pH_*i*_) and local extracellular pH (pH_*o*_). Sodium-proton exchanger 1 (NHE1, Slc9a1) eliminates protons from the cell, whereas the bicarbonate transporter, NBCn1 (Slc4a7), inwardly co-transports sodium and bicarbonate anions. With an intracellular excess of bicarbonate, this is eliminated by anion exchanger 2 (AE2, Slc4a2) (Boedtkjer et al., 2012). More recently, additional bicarbonate transporter transcripts—Slc4a3, Slc26a2, Slc26a6, Slc26a8, and Slc26a11—have been identified in rat aortic SMC *in vitro*, further increasing the complexity of intracellular pH control in VSMC (Hortells et al., 2020). In addition, carbonic anhydrases also present in VSMC can increase the concentration of bicarbonate and also alter the intracellular acid-base equilibrium. Interestingly, the inhibition of these enzymes with acetazolamide prevents the soft tissue calcification of klotho-hypomorphic mice (Leibrock et al., 2016) and in apolipoprotein E (ApoE^–/–^) mice (Yuan et al., 2019).

Furthermore, changes in pH in VSMC have been implicated in several physiological and pathological states. For example, alkaline pH_*i*_ is necessary for VSMC proliferation (Beniash et al., 2000; Boedtkjer et al., 2012), as well as for ECM remodeling and the activation of matrix metalloproteinases (Harrison et al., 1992; Stock et al., 2005). NHE1 inhibits apoptosis by increasing pH_*i*_, cell volume, and sodium content and by reducing the activity of enzymes required for apoptosis (Pedersen, 2006). Conversely, the inhibition of NHE1 or of NBCn1 should cause intracellular acidification and the reciprocal local extracellular alkalinity, consequently promoting MVC by promoting apoptosis associated with calcium nucleation (Shroff et al., 2008). This local alkalinity could also create an optimal TNAP environment for hydrolyzing PPi and organic phosphates such as phospholipids. In turn, the increased expression of TNAP seems to be one of the first steps of MVC in CKD (Hortells et al., 2017). In addition, the local alkalinity will also supersaturate the medium with respect to ACP and calcium carbonate. These examples illustrate the potential importance of pH regulation of activities in VSMC, potentially applicable to MVC pathogenesis.

### Role of Cell Transdifferentiation

Changes in local pH, TNAP overexpression and other factors, could accompany transdifferentiation of VSMC into osteo/chondroblastic-like cells. Following the detection of the bone forming gene expression in atherosclerotic lesions (Boström et al., 1993), the active role of VSMC in VC process has been extensively studied (e.g., 153–155). Osteo/chondrogenic transdifferentiation of VSMC has been mainly studied in CKD-related MVC. In these patients, the observed uremic and hyperphosphatemic conditions have tempted to the use of a simple research model accounting for a design of a fairly complete pathogenetic proposal based on direct effects of Pi as follows. The highly abundant phosphate would either permeate directly into the VSMC or it would be sensed through the sodium-phosphate cotransporters (PiT1/2), activating a signal transduction pathway resulting in a phenotypic transformation into osteochondroblast-like cells. This transformation could be mediated by the expression of Pi-induced transcription factors such as Msx2 (msh homeobox 2), Runx2 (Runt-related transcription factor 2), osterix, etc., that, in turn, would increase the expression of bone-forming proteins, such as Bmp2 (bone morphogenetic protein-2), TNAP, osteocalcin, collagen type I, etc., causing PPi depletion, increased number of nucleating sites, formation of calciprotein particles, etc., therefore facilitating, initiating, and stimulating calcification (Voelkl et al., 2019; Lee et al., 2020; Tyson et al., 2020).

Whereas VSMC transdifferentiation and the thermodynamics of CaP precipitation belong to different scientific fields, they complement each other in exploring MVC pathogenesis. While the former provides hypothesis, the later accounts for hypothesis testing. Consequently, the above outlined pathogenetical scenario has still to be considered with caution, for several reasons. Firstly, in the model or 5/6-nephrectomized rats, hyperphosphatemia is observed only after the first deposits have been formed and not before (Hortells et al., 2017), therefore, hyperphosphatemia should not be considered a necessary cause of calcification, but as an accelerator and complicating agent. Secondly, MVC may require interplay of different systemic and local factors absent in a cellular culture such as VSMC. Thirdly, thermodynamic principles suggest that calcium phosphates are not supersaturated in the normal or even CKD patients and therefore homogenous precipitation is an excluded possibility. Fourthly, in VSMC cultures, nanoparticles of calcium phosphate (or possibly calcium carbonate) rather than soluble Pi are responsible for osteo-/chondrogenic transdifferentiation. This can be easily observed by incubating VSMC cultures with high Pi concentrations in the presence of nucleating inhibitors, such as PPi, phosphonoformic acid or bisphosphonates: such nanoprecipitates are not formed and, consequently, no bone-related genes are expressed in VSMC despite the high Pi concentration (Villa-Bellosta et al., 2011; Lee et al., 2020). Fifthly, the nanoparticles do not appear to be caused by the native or transdifferentiated VSMC that would nucleate calcium heterogeneously, but rather by an alkaline pH-mediated supersaturation that causes homogeneous precipitation in media when using highly bicarbonated media in the presence of low CO_2_, along with the extremely high concentrations of Pi (Hortells et al., 2015). It is important to note that homogeneous precipitation does not occur *in vivo*. If pH is set at pH 7.4 in culture medium, no precipitates are formed, and no transdifferentiation of VSMC occurs. In fact, calcification also occurs using non-transdifferentiated, **dead** cells (Villa-Bellosta and Sorribas, 2009). Thus, the above *in vitro* experimental model represents an extreme case of biomedical research reductionism, misrepresenting the multifactorial complexity of the MVC. For example, because fluoride prevents the growth of calcium deposits *in vitro* it could be considered calcification inhibitor, yet, in contrast, fluoride promotes MVC in *in vivo* experimental rat model (5/6-nephrectomy) likely due to multitude of effects, mainly nephrotoxicity (Martín-Pardillos et al., 2014).

It has been established that osteo/chondrogenic transformation of VSMC does occur in the medial layer in MVC *in vivo*, as shown by the expression of several bone forming genes. Nevertheless, if CaP homogeneous precipitation in blood and direct Pi effect on bone forming gene expression rather effected by the calcium nanoprecipitate depositions can be excluded, s are then osteo/chondrogenic transformation could be considered a consequence of preceding calcium depositions caused for example by factors such as the local ACP supersaturation, AP overexpression and/or abundance of nucleation promoter (Hortells et al., 2017; Hruska et al., 2017), depending of the processes associated with CKD, DM, aging, or other. Therefore, we suggest, that any hypothesis of pathogenesis of MVC needs to comply with the thermodynamic principles and pass successfully the filter of energetic plausibility.

## Principles, Experimental Evidences, and Hypothesis in MVC Thermodynamics

In the final section we shall briefly outline the key principles of the fundamental laws of thermodynamics that must be obeyed in all settings and systems, evidence concerning the structure and other physical-chemical aspects of calcifications, and experimentally determined thermodynamic data that will likely require reassessments based on *in vivo* experimental data. Finally, the hypothesis of the mechanisms of MVC in the light of these guiding principles will be provided.

Firstly, the most fundamental principle relevant to any VC in any systems is that the process can commence and continue only if the conditions of supersaturation >1 are fulfilled. Thus, to define the mechanism of MVC *in vivo*, the supersaturation of the precipitating CaP compound in the milieu of the vascular medial layer must be exactly known.

Secondly, supersaturation is related to the activities of free calcium and phosphate ions that depend on concentrations and activity coefficients, which in turn vary with the ionic strength of the medium. Thus, determination of the ionic composition and activities in the vascular media is necessary to calculate true supersaturation.

Thirdly, the available experimental evidence based on the analysis of CaP calcifications in cell cultures and in arterial walls in mice indicates that the first calcium phosphate species to precipitate is ACP. Therefore, to predict the occurrence of VC the critical value of supersaturation of ACP in the arterial medial layer must be determined.

Fourthly, the experimental evidence thus far indicates that ACP is mainly composed of the PO_4_^3–^. Therefore PO_4_^3–^ is the most relevant phosphate ion species to calculate ACP supersaturation and hence to predict the likelihood of developing MVC.

Fifthly, the supersaturation is defined in relation to the thermodynamic constant, solubility product (*K*_*sp*_), of the precipitating compound. However, the available values of *K*_*sp*_ of ACP obtained *in vitro* using pH ranges unlikely to occur *in vivo* will need to be reassessed *in vivo* systems. The knowledge of *K*_*sp*_ (ACP) is critical because once ACP converts over time into HAP, calcification becomes virtually irreversible and its progression will be very difficult to stop or to reverse.

Sixthly, apart from the supersaturation, other factors also modulate the calcification process *in vivo*. These factors include primarily the presence of promoters and inhibitors at the sites of prospective calcifications. Although a number of such biomolecules has been proposed, their nature and mode of action in MVC remain to be identified. Based on the available experimental data concerning CaP precipitation we propose that promoters are likely to be large molecules containing abundant calcium-binding groups such as phosphonate, phosphate, carboxylate, sulfonate, and sulfate. These moieties are present in phosphorylated proteins, sulfated glycosaminoglycans, carboxyglutamic proteins, and phospholipids. Although the origins of these molecules within the medial layer remain to be clarified, debris of dying or dead cells represents reasonable candidates. Despite of the abundant experimental evidence concerning inhibitors, such as pyrophosphate, in blood and in the ECM with a broad potential to restrain virtually all steps of the calcification process including the nucleation of ACP, the conversion of ACP into HAP, and the crystal growth of HAP, their relevance in the MVC process remains to be clarified.

Seventhly, based on the impact of pH on the concentration of PO_4_^3–^ ions, and consequently on the supersaturation of ACP and HAP, we hypothesize that possible triggering factor for MVC calcification could be transient increases in the local pH with possible participation of the CaCO_3_ in the medial calcification process as outlined above.

## Conclusion

Calcium deposition in MVC represents a complex biological process likely involving a multitude of local and systemic factors that control the activities (rather than the concentrations) of the calcium and phosphate ions in the medial layer. The laws of thermodynamics not only delimit the number of interpretations of findings, they also assist to identify the most likely mechanisms. *In vitro* studies and early *in vivo* experimental evidence indicate that the initial precipitating phase at neutral pH is ACP, a phase that is likely undersaturated in blood, even under hyperphosphatemic conditions. Focal ACP supersaturation in the medial layer to achieve precipitating levels could be triggered by multiple factors including dramatic increase of calcium and/or phosphate activities, modest local increase in pH, imbalance between promoters and inhibitors or any combination of these.

To advance the understanding of CaP crystallization and the development of therapeutic agents preventing, inhibiting or even reversing the calcification process in MVC, research data from *in vivo* experimental protocols will be required. Compliance of these data with the laws of thermodynamics will become the litmus test of their scientific plausibility.

## Author Contributions

All authors made substantial contributions to the conception and design of the work, drafted and conducted a critical revision of the manuscript, and approved the version to be published.

## Conflict of Interest

The authors declare that the research was conducted in the absence of any commercial or financial relationships that could be construed as a potential conflict of interest. The handling editor declared a past co-authorship with one of the authors VS.
